# Accuracy of cytological examination of Tao brush endometrial sampling in diagnosing endometrial premalignancy and malignancy

**DOI:** 10.1002/ijgo.14204

**Published:** 2022-04-25

**Authors:** Antonio Raffone, Diego Raimondo, Arianna Raspollini, Alessia Oliviero, Antonio Travaglino, Angela Santoro, Federica Renzulli, Giovanni Lopez, Carlo Michele Di Maio, Paolo Casadio, Gian Franco Zannoni, Renato Seracchioli, Antonio Mollo

**Affiliations:** ^1^ Division of Gynaecology and Human Reproduction Physiopathology, Department of Medical and Surgical Sciences (DIMEC) IRCCS Azienda Ospedaliero‐Universitaria di Bologna. S. Orsola Hospital. University of Bologna Bologna Italy; ^2^ Gynecology and Obstetrics Unit, Department of Neuroscience, Reproductive Sciences and Dentistry, School of Medicine University of Naples Federico II Naples Italy; ^3^ Gynecology and Obstetrics Unit, Department of Medicine, Surgery and Dentistry "Schola Medica Salernitana" University of Salerno Baronissi Italy; ^4^ Gynecopathology and Breast Pathology Unit, Department of Woman's Health Science Agostino Gemelli University Polyclinic Rome Italy; ^5^ Anatomic Pathology Unit, Department of Advanced Biomedical Sciences, School of Medicine University of Naples Federico II Naples Italy

**Keywords:** cancer, carcinoma, diagnosis, endometrium, neoplasia, neoplasm, screening, tumor

## Abstract

Although Tao brush has become one of the most studied and used endometrial cytological samplers, concerns remain about the adequacy of the cytological sample compared with definitive histology. We aimed to assess accuracy of cytological examination from Tao brush sampling in diagnosing endometrial premalignancy and malignancy through a systematic review and meta‐analysis. Seven electronic databases were searched from January 2000 to July 2021 for all studies which allowed assessment of accuracy of Tao brush in diagnosing endometrial premalignancy and malignancy. We calculated sensitivity, specificity, positive and negative likelihood ratios (LR+ and LR−), diagnostic odds ratio (DOR) and area under the curve (AUC) on summary receiver operating characteristic (SROC) curve. Five studies with 774 patients were included. In diagnosing endometrial premalignancy and malignancy, cytological examination from Tao brush endometrial sampling showed pooled sensitivity of 0.95 (95% CI, 0.90–0.98), specificity of 0.92 (95% CI, 0.90–0.94), LR+ of 12.73 (95% CI, 3.94–41.18), LR− of 0.09 (95% CI, 0.05–0.18), DOR of 184.84 (95% CI, 24.37–1401.79), AUC of 0.9757 (standard error: 0.013). In conclusion, cytological examination from Tao brush seems to have a high diagnostic accuracy and might be proposed as both screening and diagnostic tool. However, further studies are necessary to confirm these findings.

## Synopsis

Tao brush seems to have a very high diagnostic accuracy and might be proposed as both screening and diagnostic for endometrial premalignancy and malignancy.

## 1 INTRODUCTION

Endometrial cancer (EC) is the most common gynecological neoplasia in developed countries.^1^, ^2^, ^3^, ^4^, ^5^, ^6^ In Italy, it is the third most common tumor in women between 50–69 years of age, being 4.6% of all new diagnosed neoplasia with about 86 000 new cases each year.^7^

In the last 30 years, there has been an increase in the incidence of EC,^8^ being ascribed to an aging general population and an increase in the prevalence of obesity.^9^ In 2030, it is expected that its incidence will rise by 40–50%.^10^, ^11^, ^12^ Moreover, in the last 20 years, mortality has risen even more than incidence.^8^

In clinical practice, the most common approach to diagnose EC is a transvaginal ultrasound in symptomatic women (e.g., abnormal uterine bleeding) followed by histologic examination of endometrial specimens from curettage (D&C) or hysteroscopy in patients with increased endometrial thickness.^13^

However, in order to provide an increasingly less invasive procedure and reduce the risks of infection, perforation and discomfort, endometrial cytology has been a primary focus for evaluating the endometrium.^14^, ^15^ Therefore, a large number of endometrial samplers have been proposed through the years, such as Endoflower, Tao brush, Li brush and Endocyte, with the purpose of providing both a reliable and non‐invasive endometrial sampling.^16^ Among these, after United States Food and Drug Administration approval, Tao brush has become one of the most studied and used endometrial samplers worldwide.^17^ Indeed, it allows the collection of endometrial cells without contamination from the lower genital tract and can be performed without anesthesia in outpatient settings, with minimal patient discomfort.^18^

Nevertheless, although it has shown a high rate of detecting endometrial premalignancy and malignancy, with sensitivity and specificity ranging from 91.67% to 100%, and 96% to 96.04%, respectively,^18^, ^19^ the main concern remains the adequacy of the cytological sample obtained with the Tao brush compared with definitive histology, to date. In fact, only few studies have assessed the issue and pooled data about diagnostic accuracy of Tao brush for endometrial premalignancy and malignancy are lacking.

The aim of this systematic review and meta‐analysis was to assess accuracy of cytological examination from Tao brush endometrial sampling in diagnosing endometrial premalignancy and malignancy.

## 2 MATERIALS AND METHODS

### 2.1 Study protocol

Each review stage was independently carried out by two authors following an a priori designed study protocol. Disagreements were solved by discussion with all authors.

The study followed the Synthesizing Evidence from Diagnostic Accuracy Tests (SEDATE) guidelines^20^ and the Preferred Reporting Item for Systematic Reviews and Meta‐analyses (PRISMA) statement and checklist.^21^

### 2.2 Search strategy and study selection

Seven electronic databases (i.e., Web of Sciences, Google Scholar, Scopus, MEDLINE, ClinicalTrial.gov, Cochrane Library, and EMBASE) were searched from January 2000 to July 2021 using the following text words in different combinations: “endometr*” “cancer”; “carcinoma”; “screening”; “cytolog*”; “histolog*”; “patolog*’; “tool”; “method”; “marker”; “malignancy”; “neoplasia”; “cancerous”; “premalignancy”; “precancerous”; “precancer”; “atypi*”; “hyperplasia”. References list from each eligible study was also screened for searching any studies missed during the electronic databases search.

All peer‐reviewed studies which allowed assessment of accuracy of Tao brush in diagnosing endometrial premalignancy and malignancy. A priori defined exclusion criteria were: case reports, literature review, studies in languages other than English.

### 2.3 Risk of bias within studies assessment

As suggested by Cochrane Handbook for Systematic Reviews of Diagnostic Accuracy,^22^ we adopted QUADAS‐2 (Quality Assessment of Diagnostic Accuracy Studies‐2) to estimate quality and risk of bias within the included studies.^23^ In detail, the following four domains were considered for risks of bias as depicted in the assessment tool: (1) Participant selection (i.e., if all patients were consecutively included in the study period); (2) Index test (if Tao brush endometrial sampling was carefully described and correctly performed); (3) Reference standard (if reference standard consisted of histological examination of endometrial specimen from hysterectomy); and (4) Flow and timing (if all patients underwent both index test and reference standard; if all patients were evaluated with the same tests, if the results were not affected by the latency time between index test and reference standard).

### 2.4 Data extraction

Original data from the included studies were extracted without modification. Two‐by‐two contingency tables were built for each included study based on two qualitative variables:

- Cytological examination from Tao brush endometrial sampling (Index test), dichotomized as “endometrial premalignancy and malignancy diagnosis” versus “non‐endometrial premalignancy and malignancy diagnosis”;
- Histological examination of endometrium (reference standard), dichotomized as “endometrial premalignancy and malignancy diagnosis” versus “non‐endometrial premalignancy and malignancy diagnosis”.

### 2.5 Data analysis

Cases with “endometrial premalignancy and malignancy diagnosis” from both Tao brush endometrial sampling and histological examination were considered as true positives. Cases with “non‐endometrial premalignancy and malignancy diagnosis” from both Tao brush endometrial sampling and histological examination were considered as true negatives. Cases with “endometrial premalignancy and malignancy diagnosis” from Tao brush endometrial sampling and “non‐endometrial premalignancy and malignancy diagnosis” from histological examination were considered as false positives. Cases with “non‐endometrial premalignancy and malignancy diagnosis” from Tao brush endometrial sampling and “endometrial premalignancy and malignancy diagnosis” from histological examination were considered as false negatives.

We calculated sensitivity, specificity, positive and negative likelihood ratios (LR+ and LR−), diagnostic odds ratio (DOR) and area under the curve (AUC) on summary receiver operating characteristic (SROC) curves of Tao brush endometrial sampling in diagnosing endometrial premalignancy and malignancy, as individual study and pooled estimates. Results were graphically reported on forest plots with 95% confidence interval (CI).

The accuracy of Tao brush endometrial sampling in diagnosing endometrial premalignancy and malignancy was considered as absent for AUC ≤ 0.5, low for 0.5 < AUC ≤ 0.75, moderate for 0.75 < AUC ≤ 0.9, high for 0.9 < AUC < 0.97, very high for AUC ≥ 0.97.

Statistical heterogeneity amongst the included studies was evaluated by using the Higgins I^2^ index, and judged as null for I^2^ = 0%, minimal for 0% < I^2^ ≤ 25%, low for 25 < I^2^ ≤ 50%, moderate for 50 < I^2^ ≤ 75% and high for I^2^ > 75%.

The random effect model of DerSimonian and Laird was adopted for all analyses regardless of the heterogeneity, according to the SEDATE guidelines.^20^

Data analysis was carried out adopting Review Manager 5.3 (Copenhagen: The Nordic Cochrane Centre, Cochrane Collaboration, 2014) and Meta‐DiSc version 1.4 (Clinical Biostatistics Unit, Ramon y Cajal Hospital).

## 3 RESULTS

### 3.1 Study selection and characteristics

A total of 1385 articles were identified through database searches. 26 articles remained after duplicate removal. 171 articles remained after title screening. 111 articles were evaluated for eligibility after abstracts screening. Lastly, five studies with 774 patients were included in the systematic review and meta‐analysis^18^, ^19^, ^24^, ^25^, ^26^ (Figure S1).

Among included studies, four studies were designed as observational prospective cohort studies and one study was designed as observational retrospective cohort study (Table S1). Included studies assessed 138 patients with endometrial premalignancy and malignancy, and 717 controls. From studies with extractable data, mean of patients' age, BMI and parity ranged from 31 to 87 years, from 18.3 to 58.6 kg/m^2^, and from 1.6 to 4.5, respectively. Menopause status rate ranged from 24.3% to 66.6% (Table S2). Classification of endometrial lesions by cytology and histology was shown in Table S3.

### 3.2 Risk of bias within studies assessment

In the “Patient selection” domain, two studies were judged at high risk of bias because they did not consecutively include all eligible patients in the study period,^18^, ^24^ while the remaining studies were considered at low risk.^19^, ^25^, ^26^

In the “Index test” domain, one study was considered at unclear risk of bias because the Tao brush technique was not described,^18^ while the remaining studies were considered at low risk of bias.^19^, ^24^, ^25^, ^26^

In the “Reference standard” domain, one study was considered at high risk of bias because the reference standard was D&C and not hysterectomy,^25^ three studies were considered at unclear risk of bias because the reference standard was either D&C or hysterectomy,^18^, ^19^, ^26^ while the remaining study was considered at low risk of bias.^24^

In the “flow and timing” domain, all the studies were considered at low risk of bias.

Results of risk of bias within studies assessment were graphically shown in Figure S2.

### 3.3 Diagnostic accuracy assessment

**FIGURE 1:**
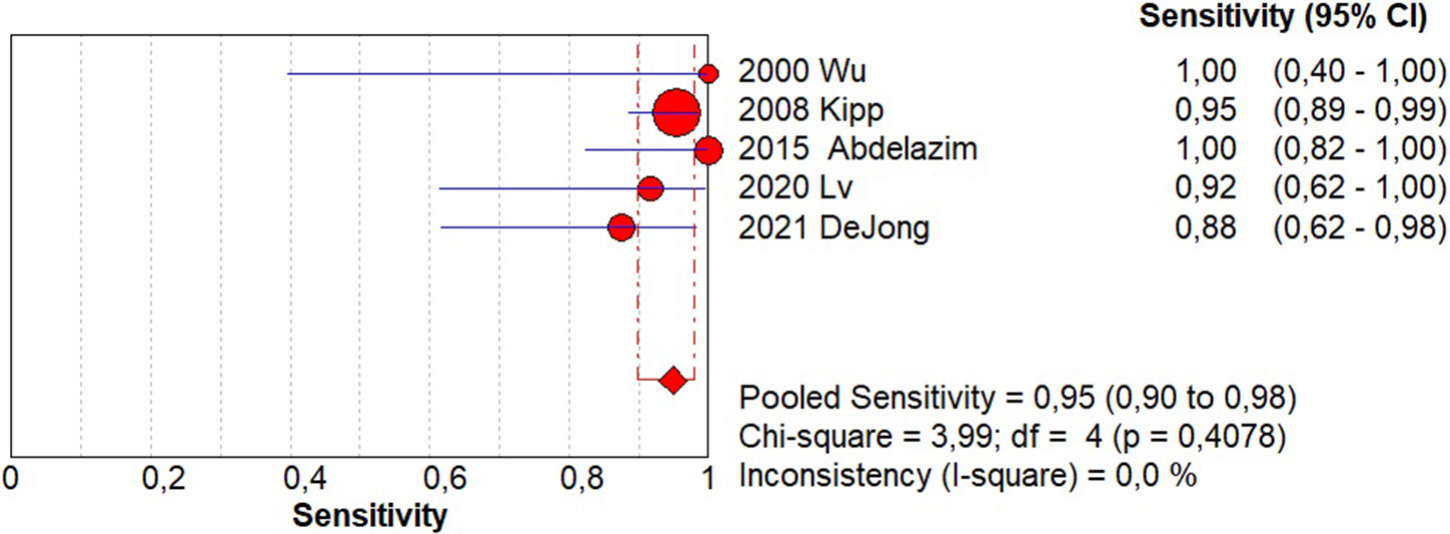
Forest plot of sensitivity of cytological examination from Tao brush endometrial sampling in diagnosing endometrial premalignancy and malignancy, as individual studies and pooled estimates

**FIGURE 2:**
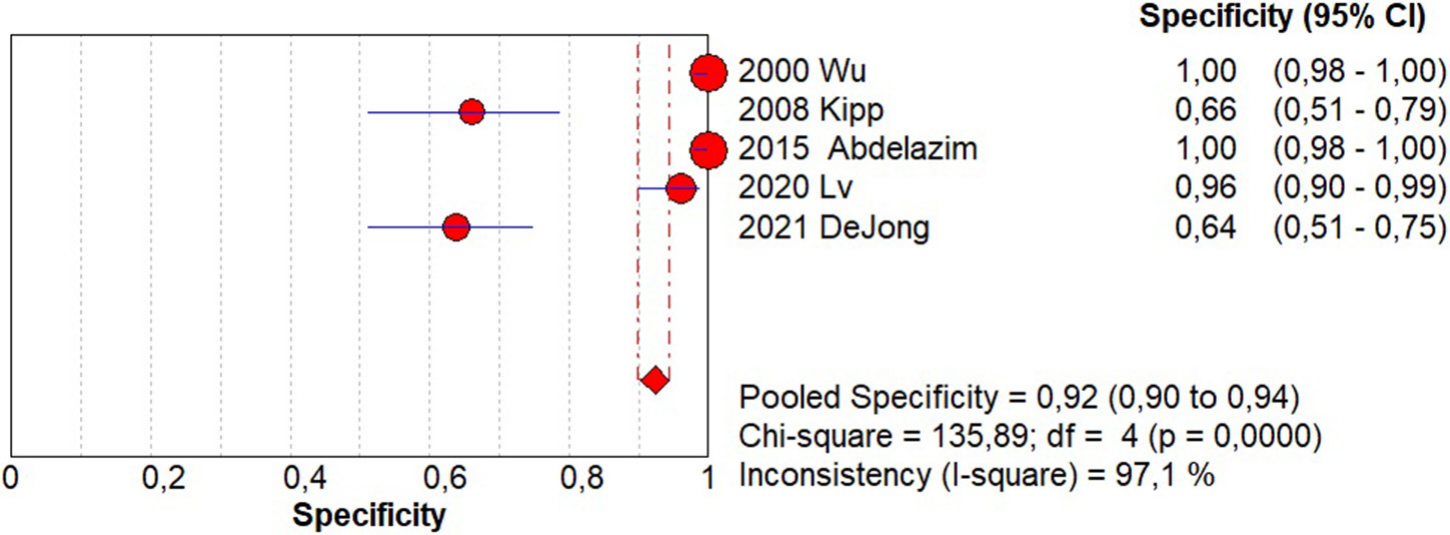
Forest plot of specificity of cytological examination from Tao brush endometrial sampling in diagnosing endometrial premalignancy and malignancy, as individual studies and pooled estimates

**FIGURE 3:**
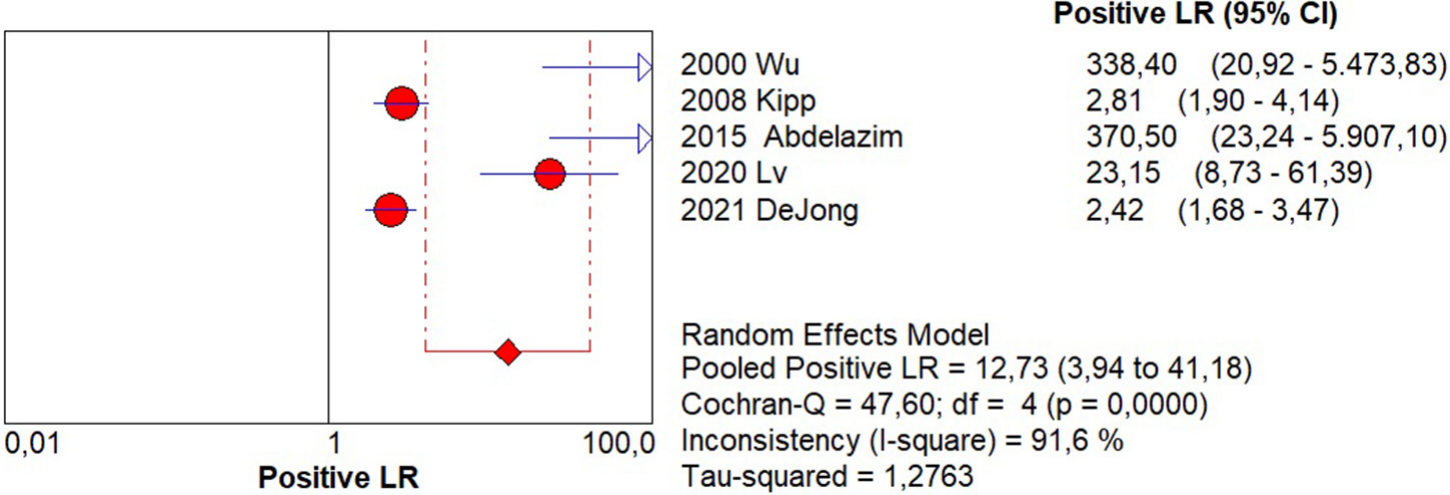
Forest plot of positive likelihood ratio of cytological examination from Tao brush endometrial sampling in diagnosing endometrial premalignancy and malignancy, as individual studies and pooled estimates

**FIGURE 4:**
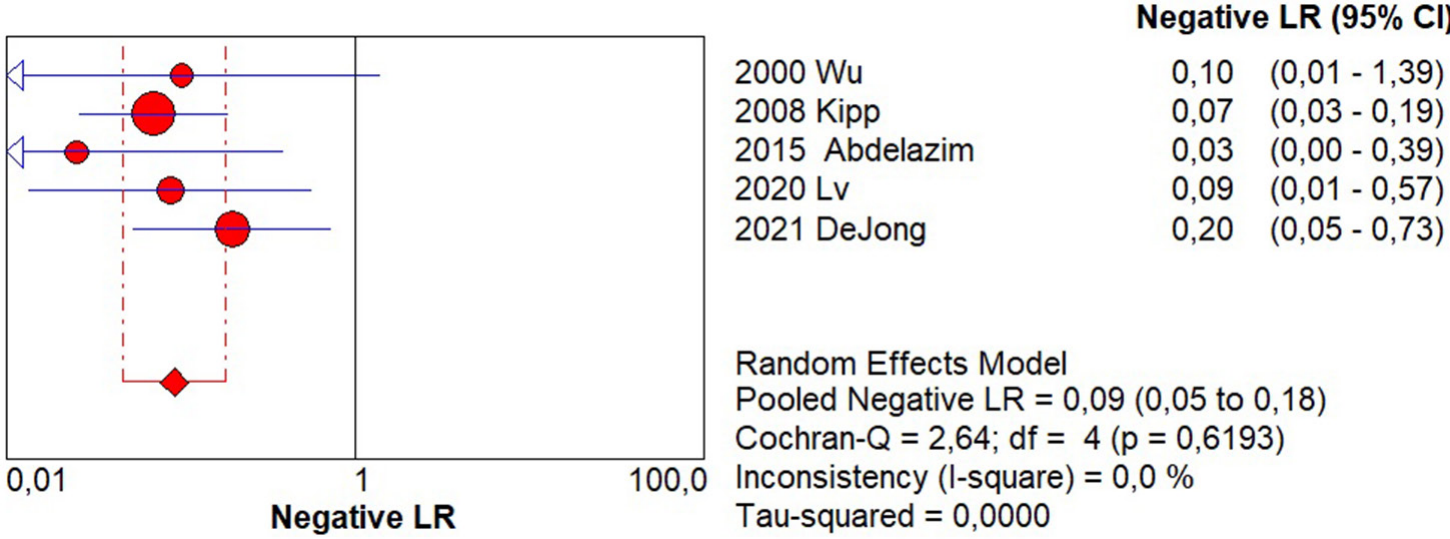
Forest plot of negative likelihood ratio of cytological examination from Tao brush endometrial sampling in diagnosing endometrial premalignancy and malignancy, as individual studies and pooled estimates

**FIGURE 5:**
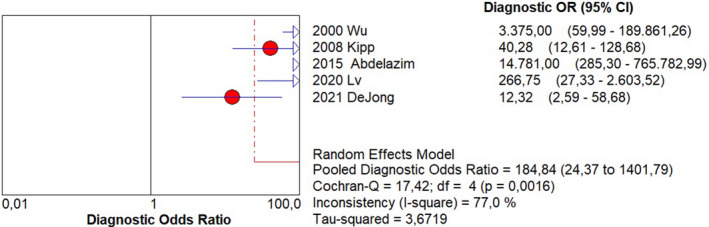
Forest plot of diagnostic odds ratio of cytological examination from Tao brush endometrial sampling in diagnosing endometrial premalignancy and malignancy, as individual studies and pooled estimates

**FIGURE 6:**
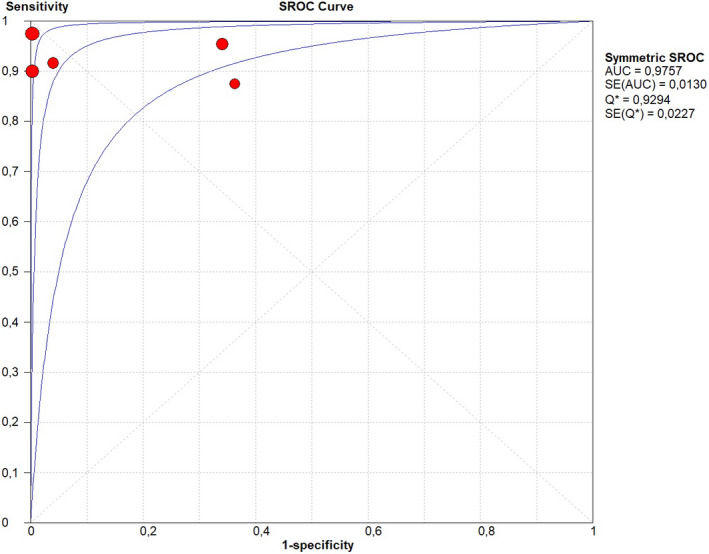
Forest plot of area under the curve (AUC) on summary receiver operating characteristic (SROC) curves of cytological examination from Tao brush endometrial sampling in diagnosing endometrial premalignancy and malignancy, as individual studies and pooled estimates

In diagnosing endometrial premalignancy and malignancy, cytological examination from Tao brush endometrial sampling showed pooled:

- Sensitivity of 0.95 (95% CI, 0.90–0.98; I^2^ = 0%; Figure 1);
- Specificity of 0.92 (95% CI, 0.90–0.94; I^2^ = 97.1%; Figure 2);
- LR+ of 12.73 (95% CI, 3.94–41.18; I^2^ = 91.6%; Figure 3);
- LR− of 0.09 (95% CI, 0.05–0.18; I^2^ = 0%; Figure 4);
- DOR of 184.84 (95% CI, 24.37–1401.79; I^2^ = 77%; Figure 5);
- AUC of 0.9757 (standard error: 0.013, Figure 6).

## 4 DISCUSSION

This study showed that cytological examination from Tao brush endometrial sampling has a very high diagnostic accuracy for endometrial premalignancy and malignancy.

In 1993, the Food and Drug Administration introduced and approved the Tao brush for general medical use.^16^, ^27^ In fact, the Tao brush is a 3.5 cm brush which is inserted at the level of the fundus through the cervical canal and then rotated 360° three to five times to collect endometrial cells. At this point, the outer sheath is pushed back to the tip, and the device is removed from the uterine cavity. The Tao brush is then cut off and immersed into cell preservation liquids and used for cytological assessment and diagnosis.^25^ Such sampling can be performed in outpatient settings without anesthetic.^24^ Due to the flexibility and design, the Tao brush has been proposed to provide a complete sampling of the endometrial cavity^28^, ^29^ and a comprehensive assessment of the epithelium because of the monolayer preparation of the sampling.^30^ This device provides some advantages when compared to endometrial sampling by D&C or hysteroscopy, such as less patient discomfort^26^, ^31^, ^32^ and more cost effectiveness.^26^, ^32^ Indeed, although only limited data are available on costs of assessment of endometrial cytology specimens, they should be similar to cervical cytology and therefore significantly lower than histology.^26^, ^33^, ^34^ As an additional advantage, the Tao brush has shown higher rates of successful insertion and tissue collection completion than other diagnostic methods of endometrial sampling.^26^

On the other hand, inter‐observer variability of endometrial cytology has been reported as a limiting factor requiring specialized pathologist training.^26^ However, DeJong et al. showed a fast learning curve for endometrial cytology.^26^ Another disadvantage of Tao brush endometrial sampling has been the difficulty in distinguishing simple hyperplasia without atypia from disordered proliferative endometrium and in diagnosing endometrial polyps.^18^, ^26^ Additionally, collecting enough endometrial cells of the uterine horns might also be a Tao brush weakness related to its round configuration. Such limitation might be overcome by another similar device for cytological sampling: the Li brush. Such a device is designed as an inverted cone similar in shape to the uterine cavity, with a theoretically higher ability in harvesting more endometrial cells in the uterine horns and fundus.

Our study shows that Tao brush endometrial sampling has a very high diagnostic accuracy for endometrial premalignancy and malignancy. Thus, due to the above‐mentioned advantages and high diagnostic accuracy, Tao brush might be proposed as a diagnostic tool for endometrial premalignancy and malignancy in women with AUB.

Alternately, Tao brush endometrial sampling might be used to triage symptomatic women to either endometrial biopsy if cytology is inconclusive or diagnosing endometrial premalignancy and malignancy, or no further investigation if cytology is clearly benign. This could spare a large proportion of women with AUB the greater discomfort associated with endometrial biopsy compared to brushing, and reduce costs for the health system.^26^, ^29^, ^31^, ^34^

Lastly, in the near future, similarly to cervical cytology, endometrial cytology might further be improved by the continuous advance in molecular testing (e.g., mutation and methylated DNA analyses, fluorescence in situ hybridization) which may complement cytological examination.^26^, ^35^, ^36^

Further studies are necessary to confirm our findings and correctly collocate endometrial cytology in the screening and diagnosis work‐up of endometrial premalignancy and malignancy.

### 4.1 Strengths and limitations

This may be the first systematic review and meta‐analysis to assess accuracy of Tao brush in diagnosing endometrial premalignancy and malignancy.

As a limitation, the overall quality of evidence is low as shown by the risk of bias within studies assessment, requiring further investigation by future well‐designed larger studies. Additionally, we were unable to assess diagnostic accuracy sub‐stratifying it based on factors which may affect endometrial cytological sampling, such as nulliparity, body mass index, menopausal status, symptoms, cycle menstrual phase, therapy, previous cesarean sections, uterine infections, and malformations.

## 5 CONCLUSION

Cytological examination from Tao brush endometrial sampling seems to have a very high diagnostic accuracy for endometrial premalignancy and malignancy. Thus, also considering its less invasive nature and greater cost effectiveness compared to endometrial histology, it might be proposed as both a screening and diagnostic tool for endometrial premalignancy and malignancy.

However, further studies are necessary to confirm these findings and correctly collocate endometrial cytology in the screening and diagnosis work‐up of endometrial premalignancy and malignancy.

### AUTHOR CONTRIBUTION

AR: study conception, study design, study methods, data extraction, data analysis, manuscript preparation. DR: study conception, study design, study methods, data analysis, manuscript preparation, methods supervision. ArR: study conception, study design, study methods, data extraction, data analysis, manuscript preparation. AO: study conception, study design, study methods, data extraction, data analysis, manuscript preparation. AT: study conception, study design, study methods, data analysis, manuscript preparation. AS: study conception, data extraction, data analysis, manuscript preparation. FR: study conception, study design, study methods, data analysis, manuscript preparation. GL: study conception, study design, study methods, data analysis, manuscript preparation. CMDM: study conception, study design, study methods, data analysis, manuscript preparation. PC: study conception, study design, methods supervision, manuscript revision, whole study supervision. GFZ: data extraction, data analysis, manuscript preparation, manuscript revision, whole study supervision. RS: study conception, study design, methods supervision, manuscript revision, whole study supervision. AM: study conception, study design, methods supervision, manuscript revision, whole study supervision. All authors approved of the final of the version to be published and agreed to be accountable for all aspects of the work in ensuring that questions related to the accuracy or integrity of any part of the work are appropriately investigated and resolved.

## ACKNOWLEDGMENTS

Open Access Funding provided by Universita degli Studi di Bologna within the CRUI‐CARE Agreement.

[Correction added on 08‐May‐2022, after first online publication: CRUI‐CARE funding statement has been added.]

## CONFLICTS OF INTEREST

The authors report no conflict of interest.

## Supporting information


Figure S1

Figure S2

Table S1

Table S2

Table S3

## Data Availability

Data sharing is not applicable to this article as no new data were created or analyzed in this study.

## References

[1] Siegel RL , Miller KD , Fuchs HE , Jemal A . Cancer Statistics, 2021. CA Cancer J Clin. 2021;71(1):7‐33. doi:10.3322/caac.21654 Epub 2021 Jan 12. Erratum in: CA Cancer J Clin. 2021 Jul;71(4):359. 33433946

[2] Travaglino A , Raffone A , Saccone G , et al. Immunohistochemical nuclear expression of β‐catenin as a surrogate of CTNNB1 exon 3 mutation in endometrial cancer. Am J Clin Pathol. 2019;151(5):529‐538. doi:10.1093/ajcp/aqy178 30715091

[3] Raffone A , Travaglino A , Mascolo M , et al. TCGA molecular groups of endometrial cancer: pooled data about prognosis. Gynecol Oncol. 2019 Nov;155(2):374‐383. doi:10.1016/j.ygyno.2019.08.019 31472940

[4] Raffone A , Travaglino A , Santoro A , et al. Accuracy of one‐step nucleic acid amplification in detecting lymph node metastases in endometrial cancer. Pathol Oncol Res. 2020;26(4):2049‐2056. doi:10.1007/s12253-019-00727-9 31444708

[5] Travaglino A , Raffone A , Saccone G , et al. Immunohistochemical predictive markers of response to conservative treatment of endometrial hyperplasia and early endometrial cancer: A systematic review. Acta Obstet Gynecol Scand. 2019;98(9):1086‐1099. doi:10.1111/aogs.13587 30793281

[6] Travaglino A , Raffone A , Mascolo M , et al. TCGA molecular subgroups in endometrial undifferentiated/dedifferentiated carcinoma. Pathol Oncol Res. 2020 Jul;26(3):1411‐1416. doi:10.1007/s12253-019-00784-0 31811476

[7] Aiom‐Artum: i numeri del cancro in Italia 2021

[8] Raffone A , Troisi J , Boccia D , et al. Metabolomics in endometrial cancer diagnosis: a systematic review. Acta Obstet Gynecol Scand. 2020;99(9):1135‐1146. doi:10.1111/aogs.13847 32180221

[9] Zhang Y , Liu H , Yang S , Zhang J , Qian L , Chen X . Overweight, obesity and endometrial cancer risk: results from a systematic review and meta‐analysis. Int J Biol Markers. 2014;29:e21‐e29. 24170556 10.5301/jbm.5000047

[10] Morice P , Leary A , Creutzberg C , Abu‐Rustum N , Darai E . Endometrial cancer. Lancet Lond Engl. 2016;387:1094‐1108. 10.1016/S0140-6736(15)00130-0 26354523

[11] Sheikh MA , Althouse AD , Freese KE , et al. USA endometrial cancer projections to 2030: should we be concerned? Future Oncol. 2014;10(16):2561e8 2561, 2568. 25531045 10.2217/fon.14.192

[12] Rahib L , Smith BD , Aizenberg R , Rosenzweig AB , Fleshman JM , Matrisian LM . Projecting cancer incidence and deaths to 2030: the unexpected burden of thyroid, liver, and pancreas cancers in the United States. Canc Res. 2014;74(11):2913e21. 10.1158/0008-5472.CAN-14-0155 24840647

[13] Braun MM , Overbeek‐Wager EA , Grumbo RJ . Diagnosis and Management of Endometrial Cancer. Am Fam Physician. 2016;93(6):468‐474. 26977831

[14] Rauf R , Shaheen A , Sadia S , et al. Outpatient endometrial biopsy with Pipelle vs diagnostic dilatation and curettage. J Ayub Med Coll Abbottabad. 2014;26(2):145‐148. 25603664

[15] Tokuda H , Nakago S , Kato H , Oishi T , Kotsuji F . Bleeding in the retroperitoneal space under the broad ligament as a result of uterine perforation after dilatation and curettage: report of a case. J Obstet Gynaecol Res. 2017;43(4):779‐782. doi:10.1111/jog.13252 28109122

[16] Du J , Li Y , Lv S , et al. Endometrial sampling devices for early diagnosis of endometrial lesions. J Cancer Res Clin Oncol. 2016;142(12):2515‐2522. doi:10.1007/s00432-016-2215-3 27515060 PMC5095161

[17] Iavazzo C , Vorgias G . Mastorakos G et al Uterobrush method in the detection of endometrial pathology. Anticancer Res. 2011;31(10):3469‐3474. 21965763

[18] Wu HH , Harshbarger KE , Berner HW , Elsheikh TM . Endometrial brush biopsy (Tao brush). Histologic diagnosis of 200 cases with complementary cytology: an accurate sampling technique for the detection of endometrial abnormalities. Am J Clin Pathol. 2000 Sep;114(3):412‐418. doi:10.1093/ajcp/114.3.412 10989642

[19] Lv S , Wang Q , Li Y , et al. A Clinical Comparative Study of Two Different Endometrial Cell Samplers for Evaluation of Endometrial Lesions by Cytopathological Diagnosis. Cancer Manag Res. 2020;12:10551‐10557. doi:10.2147/CMAR.S272755 33122953 PMC7591233

[20] Sotiriadis A , Papatheodorou SI , Martins WP . Synthesizing Evidence from Diagnostic Accuracy TEsts: the SEDATE guideline. Ultrasound Obstet Gynecol. 2016 Mar;47(3):386‐395. doi:10.1002/uog.15762 26411461

[21] Moher D , Shamseer L , Clarke M , et al. Preferred reporting items for systematic review and meta‐analysis protocols (PRISMA‐P) 2015 statement. Syst Rev. 2015;4(1):1‐4. doi:10.1186/2046-4053-4-1 25554246 PMC4320440

[22] Macaskill P , Gatsonis C , Deeks J , et al. Cochrane handbook for systematic reviews of diagnostic test accuracy. Cochrane training 2020 [cited July 10].

[23] Whiting PF , Rutjes AW , Westwood ME , et al. QUADAS‐2 Group. QUADAS‐2: a revised tool for the quality assessment of diagnostic accuracy studies. Ann Intern Med. 2011 Oct 18;155(8):529‐536. doi:10.7326/0003-4819-155-8-201110180-00009 22007046

[24] Kipp BR , Medeiros F , Campion MB , et al. Direct uterine sampling with the Tao brush sampler using a liquid‐based preparation method for the detection of endometrial cancer and atypical hyperplasia: a feasibility study. Cancer. 2008;114(4):228‐235. doi:10.1002/cncr.23636 18548528

[25] Abdelazim IA , Abdelrazak KM , Elbiaa AA , Al‐Kadi M , Yehia AH . Accuracy of endometrial sampling compared to conventional dilatation and curettage in women with abnormal uterine bleeding. Arch Gynecol Obstet. 2015;291(5):1121‐1126. doi:10.1007/s00404-014-3523-y 25367600

[26] DeJong SR , Bakkum‐Gamez JN , Clayton AC , et al. Tao brush endometrial cytology is a sensitive diagnostic tool for cancer and hyperplasia among women presenting to clinic with abnormal uterine bleeding. Cancer Med. 2021;10(20):7040‐7047. doi:10.1002/cam4.4235 34532991 PMC8525073

[27] Tao LC . Direct intrauterine sampling: the IUMC Endometrial Sampler. Diagn Cytopathol. 1997 Aug;17(2):153‐159. doi:10.1002/(sici)1097-0339(199708)17:2<153::aid-dc13>3.0.co;2-f 9258625

[28] (28) Williams AR , Brechin S , Porter AJ , Warner P , Critchley HO . Factors affecting adequacy of Pipelle and Tao Brush endometrial sampling. BJOG. 2008 Jul;115(8):1028‐1036. doi:10.1111/j.1471-0528.2008.01773.x 18651884

[29] Tanida K , Okugawa T , Sagawa N . What is the best method of detecting endometrial cancer in outpatients?‐endometrial sampling, suction curettage, endometrial cytology. Cytopathol. 2008;19(1):28‐33. doi:10.1111/j.1365-2303.2007.00509.x 17944955

[30] Sprenger E , Schwarzmann P , Kirkpatrick M , et al. The false negative rate in cervical cytology. Comparison of monolayers to conventional smears. Acta Cytol. 1996;40(1):81‐89. doi:10.1159/000333588 8604579

[31] Bagaria M , Wentzensen N , Clarke M , et al. Quantifying procedural pain associated with office gynecologic tract sampling methods. Gynecol Oncol. 2021;162(1):128‐133. doi:10.1016/j.ygyno.2021.04.033 33958213 PMC8222179

[32] Yang GC , Wan LS . Endometrial biopsy using the Tao Brush method. A study of 50 women in a general gynecologic practice. J Reprod Med. 2000;45(2):109‐114. 10710740

[33] Jin XW , Lipold L , Foucher J , et al. Cost‐effectiveness of primary HPV testing, cytology and co‐testing as cervical cancer screening for women above age 30 years. J Gen Intern Med. 2016;31(11):1338‐1344. doi:10.1007/s11606-016-3772-5 27418345 PMC5071282

[34] Kumar A . Tao Brush. J Obstet Gynaecol India. 2017;67(4):304‐305. doi:10.1007/s13224-017-1006-3 28706373 PMC5491414

[35] Bakkum‐Gamez JN , Wentzensen N , Maurer MJ , et al. Detection of endometrial cancer via molecular analysis of DNA collected with vaginal tampons. Gynecol Oncol. 2015;137(1):14‐22. doi:10.1016/j.ygyno.2015.01.552 25677060 PMC4380654

[36] Wentzensen N , Bakkum‐Gamez JN , Killian JK , et al. Discovery and validation of methylation markers for endometrial cancer. Int J Cancer. 2014;135(8):1860‐1868. doi:10.1002/ijc.28843 24623538 PMC4126846

