# Supplementary figures and images for: Accuracy of cytological examination of Tao brush endometrial sampling in diagnosing endometrial premalignancy and malignancy
Source: Int J Gynaecol Obstet. 2022 Apr 25;159(3):615–21. doi: 10.1002/ijgo.14204 (PMC9790584; doi:10.1002/ijgo.14204)

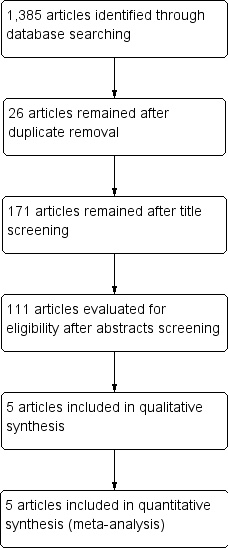

Supplement: Supplementary file 1 — Figure S1 [file IJGO-159-615-s005.jpg]

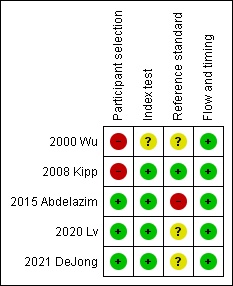

Supplement: Supplementary file 2 — Figure S2 [file IJGO-159-615-s004.zip › IJGO_14204_Supplementary figure 2a.jpg]

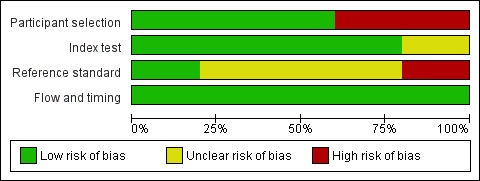

Supplement: Supplementary file 2 — Figure S2 [file IJGO-159-615-s004.zip › IJGO_14204_Supplementary figure 2b.jpg]
