# Supplementary material for: Accuracy of cytological examination of Tao brush endometrial sampling in diagnosing endometrial premalignancy and malignancy
Source: Int J Gynaecol Obstet. 2022 Apr 25;159(3):615–21. doi: 10.1002/ijgo.14204 (PMC9790584; doi:10.1002/ijgo.14204)
Supplement: Supplementary file 3 — Table S1 [file IJGO-159-615-s001.docx]

| Study | Setting | Study design | Study period | Sample size |
| --- | --- | --- | --- | --- |
| 2000 Wu | Medical Laboratories, Muncie, IN and Gynecology Associates of Muncie,PC | Prospective cohort study | 12 months | 200 |
| 2008 Kipp | Department of Laboratory Medicine and Pathology,  Mayo Clinic, Rochester, Minnesota | Retrospective cohort study | - | 139 |
| 2015 Abdelazim | Gynecology clinics of Ahmadi Hospital Kuwait Oil Company (KOC). Ahmadi, Kuwait | Prospective cohort study | 24 months | 220 |
| 2020 Lv | First Affiliated Hospital of Xi’an Jiao Tong University.  Northwest China | Prospective cohort study | 5 months | 130 |
| 2021 DeJong | Abnormal Uterine Bleeding Clinic. Women’s College Hospital. Toronto | Prospective cohort study | 30 months | 85 |
| TOTAL |  |  |  | **774** |

**Supplementary Table 1.** Characteristics of included studies.

**-:** not available
