# Supplementary material for: Accuracy of cytological examination of Tao brush endometrial sampling in diagnosing endometrial premalignancy and malignancy
Source: Int J Gynaecol Obstet. 2022 Apr 25;159(3):615–21. doi: 10.1002/ijgo.14204 (PMC9790584; doi:10.1002/ijgo.14204)
Supplement: Supplementary file 4 — Table S2 [file IJGO-159-615-s003.docx]

**Supplementary Table 2.** Patients’ characteristics.

| Study | Age [years] mean ± SD or median (range) | BMI [kg/m^2^], mean ± SD (range) | Menopausal status [years],  % | Menarca, [years], mean ± SD (range) | Parity, mean ± SD |
| --- | --- | --- | --- | --- | --- |
| 2000 Wu | - | - | - | - | - |
| 2008 Kipp | 60  (31-87) | - | - | - | - |
| 2015 Abdelazim | 48,6 ± 5.6  (40-51) | - | - | 11.8 ± 2.1 (11-15) | 4.5 ± 3.2 |
| 2020 LV | Cancer group: 54.9 ± 8.3;  Non cancer group: 47.0 ± 9.6 | Cancer group: 23.3 ± 2.8;  Non cancer group: 23.6 ± 3.4 | Cancer group: 66.6;  Non cancer group: 24.3 | Cancer group: 14.5 ± 2;  Non cancer group:  14.6 ± 1.8 | Cancer group 1.8 ± 1.1;  Non cancer group:  1.6 ± 0.9 |
| 2021 DeJong | 55 ± 7.6  (45.1- 78.5) | 31.7 ± 8  (18.3-58.6) | 60 | - | - |

**-:** not available; **BMI:** body mass index; **SD:** standard deviation
