# Supplementary material for: Accuracy of cytological examination of Tao brush endometrial sampling in diagnosing endometrial premalignancy and malignancy
Source: Int J Gynaecol Obstet. 2022 Apr 25;159(3):615–21. doi: 10.1002/ijgo.14204 (PMC9790584; doi:10.1002/ijgo.14204)
Supplement: Supplementary file 5 — Table S3 [file IJGO-159-615-s002.docx]

**Supplementary Table 3.** Classification of endometrial lesions by cytology and histology.

| Study | Cytology | | Histology | |
| --- | --- | --- | --- | --- |
|  | **Benign** | **Malignant** | **Benign** | **Malignant** |
| 2000 Wu | 187 | 4 | 187 | 4 |
| 2008 Kipp | 37 | 100 | 50 | 87 |
| 2015 Abdelazim | 189 | 19 | 189 | 19 |
| 2020 LV | 98 | 15 | 101 | 12 |
| 2021 DeJong | 46 | 39 | 69 | 16 |
| TOTAL | 557 | 177 | 596 | 138 |

Benign endometrial lesions included: proliferative endometrium, secretory endometrium, endometrial, hyperplasia without atypia, endometritis or endometrial polyp

Malignant endometrial lesions included: endometrial hyperplasia with atypia, endometrial carcinoma
